# Waterpipe smoking among health sciences university students in Iran: perceptions, practices and patterns of use

**DOI:** 10.1186/1756-0500-4-496

**Published:** 2011-11-16

**Authors:** Nasim Ghafouri, Jan D Hirsch, Gholamreza Heydari, Candis M Morello, Grace M Kuo, Renu F Singh

**Affiliations:** 1 Skaggs School of Pharmacy and Pharmaceutical Sciences, University of California, San Diego, CA, USA; 2 Veterans Affairs San Diego Healthcare System, San Diego, CA, USA; 3 Shahid Beheshti University of Medical Sciences, Tehran, Iran; 4 Tobacco Prevention and Control Research Center, National Research Institute of Tuberculosis & Lung Disease, Tehran, Iran; 5 School of Medicine Department of Family and Preventive Medicine, University of California, San Diego, CA, USA

## Abstract

**Background:**

In recent years waterpipe smoking has become a popular practice amongst young adults in eastern Mediterranean countries, including Iran. The aim of this study was to assess waterpipe smoking perceptions and practices among first-year health sciences university students in Iran and to identify factors associated with the initiation and maintenance of waterpipe use in this population.

**Results:**

Out of 371 first-year health sciences students surveyed, 358 eight students completed a self-administered questionnaire in the classrooms describing their use and perceptions towards waterpipe smoking. Two hundred and ninety six responders met study inclusion criteria. Waterpipe smoking was common among first-year health sciences university students, with 51% of students indicating they were current waterpipe smokers. Women were smoking waterpipes almost as frequently as men (48% versus 52%, respectively). The majority of waterpipe smokers (75.5%) indicated that the fun and social aspect of waterpipe use was the main motivating factor for them to continue smoking. Of waterpipe smokers, 55.3% were occasional smokers, using waterpipes once a month or less, while 44.7% were frequent smokers, using waterpipes more than once a month. A large number of frequent waterpipe smokers perceived that waterpipe smoking was a healthier way to use tobacco (40.6%) while only 20.6% thought it was addictive. Compared to occasional smokers, significantly more frequent smokers reported waterpipe smoking was relaxing (62.5% vs. 26.2%, *p *= 0.002), energizing (48.5% vs. 11.4%, *p *= 0.001), a part of their culture (58.8% vs. 34.1%, *p *= 0.04), and the healthiest way to use tobacco (40.6% vs. 11.1%, *p *= 0.005).

**Conclusions:**

Social and recreational use of waterpipes is widespread among first-year health sciences university students in Iran. Women and men were almost equally likely to be current waterpipe users. Public health initiatives to combat the increasing use of waterpipes among university students in Iran must consider the equal gender distribution and its perception by many waterpipe smokers as being a healthier and non-addictive way to use tobacco.

## Background

Waterpipe smoking originated nearly four centuries ago in ancient Persia and India [1] and involves the passage of tobacco smoke through water before inhalation via a long pipe. In recent years, waterpipe smoking (also known as ghelyan, hookah, shisha, narghile) has seen a significant rise in popularity in Iran and other Eastern Mediterranean Region (EMR) countries [2-6].

In Iran, the usual age of initiating any type of tobacco smoking is between 13-20 years [7-9] with youth being the fastest growing segment of the waterpipe smoking trend. While cigarette smoking prevalence has been estimated to be 3% among adolescents aged 13-15 years in Iran, from 2003 to 2005 waterpipe smoking increased from 35.5% to 40.9% in males, and from 19.7% to 26.1% in females aged 10-18 years [7,10]. Waterpipe smoking is becoming more prevalent among university students in EMR countries and by students of Middle Eastern descent living in Western countries [11,12]. Among Iranian university students, 11.5% of females and 28.7% of males have been reported to smoke waterpipes, compared to 2.5% of females and 18.3% of males who smoke cigarettes [13]. The studies discussed above did not report the frequency of waterpipe use in respondents.

A meta-analysis of six studies reported that the daily use of a waterpipe with tobacco produced nicotine absorption rates equivalent to smoking 10 cigarettes a day [14]. A single 30-minute waterpipe smoking episode produces higher exposure to tobacco and carbon monoxide than a single cigarette [15]. As a result, regular waterpipe smokers are at risk for nicotine dependence. Waterpipe smoking has been associated with an increased risk of lung cancer, respiratory illness, low birthweight, and periodontal disease [16]. The common practice of sharing waterpipe mouthpieces may also increase the risk of transmitting tuberculosis [17] and orally transmitted viral illnesses. Waterpipe smoking has been associated with statistically lower health-related quality of life scores than non-smokers specifically related to physical function, bodily pain, general health, mental health, vitality, and social function [18]. The World Health Organization (WHO) has warned that waterpipe smoking may pose the same health risks as cigarettes, and has urged more research to better understand the link between waterpipe use and various illnesses [19].

As institutions of higher learning, universities can be expected to have a higher number of well-educated, well-informed young people in attendance. There have been few studies in Iran examining whether students studying in health sciences have different behaviors towards waterpipe use. We sought to study attitudes and practices of waterpipe use among first-year health sciences university students taking science courses in Iran, and factors motivating their initiation and maintenance.

## Methods

### Study design and data collection

This study was a cross-sectional survey at a health sciences university in Iran. The survey instrument (see Additional file 1) was developed from a literature review and questions adapted from previously published waterpipe smoking studies [6,20]. The survey was initially developed in English and subsequently translated into Farsi by two Farsi-speaking primary investigators (NG, GH). The survey was reviewed by several Farsi-speaking laypeople to ensure consistency of meaning prior to the start of the study. The study was conducted at Shahid Beheshti University of Medical Sciences (SBU), Tehran, Iran. The student population at SBU was 2250; 630 were first-year graduate students in health sciences degree programs (e.g., Medicine, Dental, Pharmacy, or Health). The surveys were distributed to 371 of the first-year graduate students taking physics, chemistry, biology, and research methods classes over a span of 2 weeks in December 2007. The principal investigators (NG, GH) in Iran appointed several individuals to aid in survey administration: two were course instructors at SBU and two were university students. Students were verbally informed of the scope of the survey and were provided with a waiver of consent information sheet prior to completing the survey. In total, 358 students (a 96.5% response rate) completed the anonymous survey without assistance from the course instructors or other students.

Inclusion criteria for our study required each respondent to be a first-year health sciences student, aged between 18 and 30 years, able to read Farsi, and provide consent.

### Questions and variables

Using a self-administered survey, the respondents were asked information on whether they had ever smoked waterpipes, and if so, at what age they started, what attracted them to smoke waterpipes, and with whom they smoked the first time they used a waterpipe. In addition, several questions asked if they currently smoked waterpipes, and if so, their frequency of smoking, their family's feelings regarding their waterpipe use, and factors motivating them to continue smoking. Respondents were also asked to report their age and gender.

No subject identifiers were collected and all data were anonymous. Data for respondents who met the inclusion criteria (SBU first-year graduate health sciences student, age between 18 and 30 years, able to read Farsi, consent provided) were analyzed. After survey completion, all participants received educational brochures highlighting the risks, misconceptions, and health hazards of both waterpipe and cigarette smoking. The study was approved by the institutional review boards at the Shahid Beheshti University of Medical Sciences (SBU), Tehran, Iran and the University of California, San Diego (UCSD).

### Definitions

We defined anyone who had ever tried waterpipes, regardless of whether they smoked waterpipes currently, as ever waterpipe smokers. We defined current waterpipe smokers as anyone who indicated on their survey that they smoked waterpipes currently. Of the current waterpipe smoker group, we defined occasional smokers as those using waterpipes less than or equal to once a month, and frequent smokers as using waterpipes more than once a month.

### Data analysis

Completed survey data were entered into an Excel datasheet and analyzed using STATA version 10.0. Measures of central tendency were expressed as mean ± standard deviation (SD). Frequency data were expressed as number of cases for each response category and percentage response. Fisher's Exact test was used to assess differences in smoking attitudes between occasional and frequent waterpipe smokers, with an a priori alpha = 0.05 level of significance.

## Results

Three hundred and seventy one surveys were distributed to first-year health science students. A total of 358 surveys were collected, a response rate of 96.5%. Sixty two participants did not meet inclusion criteria (10 were younger than 18 years, 14 were older than 30 years, and 38 specified no age), thus 296 (82.7%) were included in the final analysis. The average respondent age was 22 years (SD ± 3.0); 53% were female. A total of 169 participants answered the question regarding current waterpipe use, with 51% indicating they were current waterpipe smokers. Out of these respondents, 52% were men and 48% were women. The waterpipe smoking habits in current users included 55.3% occasional smokers and 44.7% frequent smokers.

**Figure 1:**
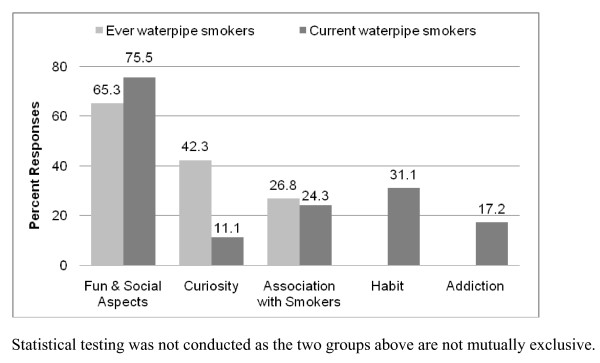
**Top-rated factors motivating waterpipe initiation and continuation**.

Ever waterpipe smokers and current waterpipe smokers reported initiating waterpipe smoking in their mid-teens (17.1 and 16.9 years, respectively), and smoked for the first time mostly with friends (50.9% and 55.8%, respectively) or family members (44.6% and 43%, respectively). Over one-third of each group had family members who smoked waterpipes at home (39.2% and 44.6%, respectively). The fun and social aspects of waterpipe smoking were cited as the primary reason for initiating and continuing waterpipe use (Figure 1). Among ever waterpipe smokers, 42.3% had tried waterpipes out of curiosity. In the current waterpipe smokers cohort, 31.1% reported that waterpipe use had become a habit for them but only 17.2% felt that it was addicting. Statistical testing was not conducted as the two groups were not mutually exclusive.

Age, gender, and smoking practices were similar between the occasional and frequent waterpipe smoker groups. Occasional and frequent smokers reported an increase in smoking during the holidays (44.4% and 63.6%, respectively; *p *= 0.11) and during the summer months (58.1% and 81.3%, respectively; *p *= 0.05), whereas fewer students in this cohort reported stress or exams as a cause of increased smoking frequency (15% and 34.4%, respectively; *p *= 0.09). A third of students in both groups reported sharing their waterpipe mouthpiece with other smokers (34% vs *32.4%, p = 1.0*). Approximately three-quarters (78.7% vs 73%, *p *= 0.61) of occasional and frequent waterpipe smokers smoked with a friend, whereas one-fourth (25.5% vs 24.3%, *p *= 0.99) smoked with family members. When asked about their family's feelings towards respondent's waterpipe use (accepting, no specific reaction, or non-accepting), 50% of occasional and 44.1% of frequent waterpipe smokers reported that family members were non-accepting of their smoking behavior *(p *= 0.044).

Perceptions towards waterpipe smoking varied between occasional and frequent waterpipe users as demonstrated in Additional file 2, Table 1. A greater number of frequent waterpipe smokers perceived that waterpipe smoking was the healthiest way to use tobacco than occasional waterpipe users. Frequent waterpipe smokers were more likely than occasional waterpipe smokers to indicate that waterpipe smoking was relaxing, gave them energy, and was a part of their culture. While a large majority (73.3% and 67.7%) of both groups felt that waterpipe smoking was dangerous for their health, paradoxically only 22.2% and 20.6% in the occasional and frequent group, respectively, felt that it was addicting.

## Discussion

Waterpipe smoking prevalence has been reported to be high among university students in EMR countries [12]. The results of the present study show that waterpipe smoking is common among young adults (with similar frequencies reported by men and women) studying health sciences at a university in Iran. A systematic review of prevalence of waterpipe smoking from 38 studies found rates of current waterpipe smoking in university students to be 15-28% in EMR countries, 33% in the South Asia region, 10% in the Americas, and 8% in Europe [12]. However, no studies from Iran were included in this review. Waterpipe smoking among university students in Isfahan, Iran, has been reported to be 28.7% in men and 11.5% in women [13]. Self-administered questionnaires in Iranian dental students reveal that 23% are current cigarette, pipe or waterpipe users [21]. A study of university students in South Iran found a prevalence of 18.7% for students who had used waterpipes in the previous 30 days [22] whereas state university students in Iran reported 40.3% waterpipe use [23]. The latter study included 4433 undergraduate students from 7 universities and included universities located in Tehran, Iran [23]. Our study shows that among health sciences university students in Tehran, 51% report occasional or frequent waterpipe use. Our results confirm the higher rates of waterpipe use in university students reported by others in Iran [23] compared to university students from other EMR countries [12]. Students surveyed in our study were first-year graduate students enrolled in a health sciences degree program. While it could be argued that health sciences curriculums are demanding and stressful, most current waterpipe smokers in our study cited social and fun aspects as the primary reason for their continuing use of waterpipes. Few cited stress of university or exams as a motivator for smoking. However, it is concerning that the percent of waterpipe smokers is so high at a health sciences university where students presumably have greater knowledge of the risks and dangers posed by tobacco use and will be entering a career in health care.

Despite the high waterpipe use observed in our study, the degree of waterpipe smoking intensity is likely lower. Occasional waterpipe smokers accounted for slightly more than half (55.3%) of current waterpipe users. Students who were frequent waterpipe smokers (44.7%) were defined as using waterpipes as more than once a month and included students who used waterpipes daily, a few times a week, weekly, or once every 2 weeks. However, a study of Iranian dental students reported that only 7% smoked waterpipes on a daily basis [21].

Contrary to previous studies in this population, ours shows a similar number of women (48%) and men (52%) who are occasional or frequent waterpipe smokers. An increasing frequency of female smoking has been demonstrated, especially among youth [8]. While societal taboos exist against cigarette smoking by women in Iran and other EMR countries, attitudes towards waterpipes smoking are more lax and acceptable in a social and cultural context [4,20,24-26]. The high frequency of waterpipe use amongst female students seen in our cohort is mirroring a trend of independence and equal rights that young women are seeking in Iran. Over the last decade, young Iranian women have been enrolling in universities in increasing numbers and currently comprise over half of its university students [27]. Tehran is a diverse, densely populated capital city where many young men and women seek a modern lifestyle. Shahid Beheshti University of Medical Sciences (SBU) is the second largest university in Iran, offering a number of medical and non-medical health sciences curriculums and accepting high-achieving students from different socioeconomic backgrounds. While the academic rigor of SBU is likely to be similar to other health science universities in Iran, its location in its capital city and the large number of women students in attendance may be a contributing factor to the high waterpipe smoking frequency observed in this study. Whether this waterpipe smoking frequency is a reflection of a larger trend occurring in well educated young women in Tehran is a subject for future studies. A recent study reported similarly high rates of waterpipe smoking in university students in Iran (including universities from Tehran), suggesting that higher waterpipe smoking rates are observed in Tehran than in other parts of the country [23]. The authors reported that waterpipe use was more common in men (OR 0.42), and reported the odds ratio for waterpipe smoking to be 26.51 for students from universities located in the west of Iran, including Tehran [23]. This lends support to our findings as our research was conducted at a health sciences university in Tehran.

Waterpipe smoking was perceived as an enjoyable social activity among friends. Indeed, this was rated as the primary motivator behind both initial experimentation and continuation of waterpipe smoking in our cohort. Respondents in our study reported that their waterpipe smoking increased during the holidays and summer months, and most smoked in the company of friends and family, consistent with previously published findings [5,9,13,25]. The role of family in waterpipe smoking appears complex and contradictory. Family members are often waterpipe smokers themselves and are frequently part of the introductory waterpipe smoking experience [2,3]. However, the apparent initial support of waterpipe smoking by the family may decrease over time, since in our study approximately half of occasional waterpipe smokers reported their family members disapproved of their continued waterpipe smoking habits. Acknowledging and understanding the connection of family in introducing and fostering waterpipe smoking is an essential element in developing strategies to curtail and reduce its use in Iran.

Our study examined student perceptions of waterpipe use. More than half of frequent waterpipe smokers perceived waterpipe smoking to be relaxing, energizing, and a part of their culture in our study. However, when questioned about the dangers of waterpipe smoking, the majority of frequent smokers reported that while they believed waterpipe smoking to be hazardous to their health, they also erroneously believed waterpipes were the healthiest choice among tobacco products. This perception is particularly worrisome as a growing body of literature reveals the negative healthcare effects of waterpipe smoking on exposure to tobacco, carbon monoxide, addiction, and second-hand smoke exposure [12,28-30]. For example, waterpipe smoking has been associated with predicting progression to regular cigarette use in Danish adolescents [31].

The study was limited in that surveys were distributed to a proportion of first-year health sciences students, rather than all first-year health science students, possibly creating a selection bias. Students completed the survey in a paper format without assistance from trained personnel. The survey involved several conditional questions asking participants to either skip or continue with subsequent questions depending on their previous responses. However, some students interpreted these directions incorrectly and demonstrated skip patterns that were inappropriate, thus creating missing data and reducing our usable sample size. A number of questions were not answered by all respondents, which can be observed in Additional file 2, Table 1, which may have introduced bias in our findings.

This, in combination with the study's convenience sampling, may have contributed to the high frequency of waterpipe smoking that we observed. Furthermore, while the survey questions were adapted from previous studies on waterpipe smoking, the final survey instrument was not validated prior to distribution. In contrast to cigarette smoking, there is currently no consensus in the research community regarding what defines an occasional or frequent waterpipe user. We defined occasional smokers as those using waterpipes less than or equal to once a month, and frequent smokers as those using waterpipes more than once a month, while other investigators have suggested recording waterpipe patterns using categories such as 'daily', 'weekly', or 'monthly'[32]. A consistent definition is needed to allow comparison of results across study populations and outcomes of interventions to reduce waterpipe smoking.

The findings of this study reflect the responses of students at a health sciences university in Tehran, Iran, and conclusions from our study may be different than those of the general waterpipe smoker population in Iran. However, while cigarette smoking is more prevalent in lower socioeconomic and less educated populations and occurs predominantly among males in Iran [8], our study results confirm findings from similar studies [2,13,21-23] that waterpipe smoking has developed a surprisingly strong user base within highly educated young men and women. This suggests that waterpipe smoking has become a socially acceptable practice within this demographic.

Health promotion strategies have been developed for the EMR that urge member countries to be proactive in addressing emerging health risk factors, including smoking cessation [1]. Strategies to combat the increasing use of waterpipes among youth and young adults need to be included in these efforts. Public health authorities in Iran need to develop educational programs that reach youth in schools and young adults in universities, particularly women. Since family members were involved with the introduction of waterpipes in almost half of current waterpipe users in this study, messages targeting and involving families should be an essential component of any educational program seeking to reduce the widespread practice of waterpipe smoking in Iran. Furthermore, teachers and faculty at schools and universities should be engaged to include tobacco and waterpipe prevention programs in their curriculum. Faculty at health sciences university campuses in particular should ensure that their curriculums include the dangers and addicting qualities of tobacco use, including waterpipe smoking, and discuss prevention and treatment of tobacco addiction. University administrators should strongly discourage the use of all forms of tobacco on and off their campuses.

## Conclusions

Our study found 51% of first-year health sciences university students to be current waterpipe smokers in Tehran, Iran. This highlights the lack of awareness of the negative consequences of waterpipe smoking even among well-educated health professional trainees. Public health campaigns need to educate and reach both genders, youth, university students, and families. Universities should be engaged in educating their students against the health risks of all tobacco use, including waterpipe smoking.

## Competing interests

The authors declare that they have no competing interests.

## Authors' contributions

NG conceived the study, participated in its design, coordination, performed statistical analyses, and co-drafted the manuscript

JDH participated in the design of the study, performed statistical analysis and helped with the manuscript draft

GH assisted with study design and coordination and helped with the manuscript draft

CMM participated in the design of the study and analysis of the study results and helped with the manuscript draft

GMK participated in analysis of the study results and helped with the manuscript draft

RFS participated in the study design and coordination and co-drafted the manuscript

All authors read and approved the final manuscript.

## Funding

None

## Supplementary Material

Additional file 1 **WPS survey**. Click here for file

Additional file 2 **Table 1 - Attitudes of waterpipe smoking between occasional and frequent waterpipe smokers**. Click here for file
