# Supplementary material for: Waterpipe smoking among health sciences university students in Iran: perceptions, practices and patterns of use
Source: BMC Res Notes. 2011 Nov 16;4:496. doi: 10.1186/1756-0500-4-496 (PMC3279519; doi:10.1186/1756-0500-4-496)
Supplement: Additional file 1 — WPS survey. [file 1756-0500-4-496-S1.DOC]

WPS Survey Survey Number:__________

Location:________________

Please read and answer the following questions by marking an “X” in the appropriate box.

1. Gender: ⁪□^1^ Male ⁪□^2^ Female

2. Age: _________ years

3. Have you ever smoked a waterpipe (ghalyoun; hookah)?

⁪□^1^ Yes Continue with question 4.

⁪□^2^ No Proceed to question 26.

4. At what age did you first smoke a waterpipe? ________ years

5. The first time you smoked a waterpipe, you were:

□^1^ Alone ⁪

□^2^ With a friend(s) ⁪

□^3^ With family ⁪

□^4^ Other (specify):_________

□^5^ I don’t remember

6. What factor(s) attracted you to smoking waterpipes your first time? Please rank the following

factors on a scale of 0-5, 0 meaning that factor had no role, and 5 meaning that factor played a

large role in your desire to smoke.

__^1^Curiosity

__^2^Fun/Social aspects

__^3^ Stress

__^4^ Associating with people who smoke waterpipes

__^5^Anger

__^6^Imitation

⁪ __^7^ I was offered

⁪ __^8^Other (specify): __________________

7. Do any of your family members smoke waterpipes at home? ⁪□^1^ Yes □^2^ No

8. Do you smoke waterpipes currently?

⁪□^1^ Yes Continue with question 9.

⁪□^2^ No Proceed to question 26.

9. How often do you smoke waterpipes? (Select ONE)

□^1^Daily

□^2^Once a week

⁪ □^3^Once every 2 weeks

⁪□^4^Once a month

⁪□^5^A few times a week

10. For approximately how long have you been smoking this amount?

□^1^Less than one month

□^2^Between one month and one year

⁪ □^3^More than one year

11. Indicate whether there is an increase, decrease, or no change in your frequency of waterpipe use

under the following circumstances:

a) Stress/Exams ⁪□^1^Increase ⁪□^2^ Decrease ⁪□^3^ No change

b) Holidays ⁪□^1^ Increase ⁪□^2^ Decrease ⁪□^3^ No change

c) Summer ⁪□^1^ Increase ⁪□^2^ Decrease ⁪□^3^ No change

12. Currently, what factor(s) motivate you to continue smoking waterpipes? Please rank the

following factors on a scale of 0-5, 0 meaning that factor has no role, and 5 meaning that factor

plays a large role in your desire to continue smoking.

__^1^Curiosity

___^2^Stress

__^3^Imitation

__^4^Anger

⁪ __^5^ I was offered

__^6^ Addiction

__^7^ Fun/Social aspects

__^8^  Habit

__^9^ Associating with people who smoked waterpipes

⁪ __^10^Other (specify): __________________

13. Currently, you generally smoke waterpipes when you are:

⁪□^1^ Alone ⁪□^2^ With a friend(s) ⁪□^3^ With family □^4^ Other (specify):_________

14. Where do you usually smoke waterpipes?

⁪□^1^ Home

⁪□^2^ Friend/family’s residence

⁪□^3^ Café/Restaurant

⁪□^4^ No particular place

⁪□^5^ Other (specify):________________

15. Do you share a waterpipe mouthpiece with others? ⁪□^1^ Yes ⁪□^2^ No

If so, with whom do you most commonly share the mouthpiece?

⁪□^1^ Friend(s)

⁪□^2^ Family

⁪□^3^ Fellow smoker

⁪□^4^ Other (specify): _____________

16. Waterpipe smoking is a healthy way to use tobacco.

⁪□^1^ I agree ⁪□^2^ I have no opinion ⁪□^3^ I disagree

17. Smoking waterpipes makes one look attractive.

⁪□^1^ I agree ⁪□^2^ I have no opinion ⁪□^3^ I disagree

18. Waterpipe smoking is dangerous to ones health.

⁪□^1^ I agree ⁪□^2^ I have no opinion ⁪□^3^ I disagree

19. Smoking waterpipes is relaxing.

⁪□^1^ I agree ⁪□^2^ I have no opinion ⁪□^3^ I disagree

20. Smoking waterpipes gives me energy.

⁪□^1^ I agree ⁪□^2^ I have no opinion ⁪□^3^ I disagree

21. Waterpipes have a pleasant taste and smell.

⁪□^1^ I agree ⁪□^2^ I have no opinion ⁪□^3^ I disagree

22. Smoking waterpipes is a part of my culture.

⁪□^1^ I agree ⁪□^2^ I have no opinion ⁪□^3^ I disagree

23. Waterpipe smoking is addictive.

⁪□^1^ I agree ⁪□^2^ I have no opinion ⁪□^3^ I disagree

24. What effect does the water in a waterpipe have on its use?

□^1^ It filters toxic substances.

□^2^ It neutralizes carcinogenic substances.

⁪ □^3^ It absorbs carbon monoxide.

□^4^ It humidifies the smoke, and thus increases the risk of developing health problems.

25. What is your family’s feelings regarding your waterpipe use? (Select ONE)

⁪□^1^ Friendly/Accepting

⁪□^2^ Normal/No specific reaction

⁪□^3^ Not Friendly/Non-accepting

26. Do you have experience smoking cigarettes?

⁪□^1^ Yes Continue to question 27.

⁪□^2^ No Proceed to question 38.

27. At what age did you first smoke a cigarette? ________ years

28. Do you smoke cigarettes currently?

⁪□^1^ Yes Continue to question 29.

⁪□^2^ No Proceed to question 38.

29. How many cigarettes do you smoke daily? ______________

30. Smoking cigarettes makes one look attractive.

⁪□^1^ I agree ⁪□^2^ I have no opinion ⁪□^3^ I disagree

31. Cigarette smoking is dangerous to ones health.

⁪□^1^ I agree ⁪□^2^ I have no opinion ⁪□^3^ I disagree

32. Smoking cigarettes is relaxing.

⁪□^1^ I agree ⁪□^2^ I have no opinion ⁪□^3^ I disagree

33. Smoking cigarettes gives me energy.

⁪□^1^ I agree ⁪□^2^ I have no opinion ⁪□^3^ I disagree

34. Cigarettes have a pleasant taste and smell.

⁪□^1^ I agree ⁪□^2^ I have no opinion ⁪□^3^ I disagree

35. Smoking cigarettes is a part of my culture.

⁪□^1^ I agree ⁪□^2^ I have no opinion ⁪□^3^ I disagree

36. Cigarette smoking is addictive.

⁪□^1^ I agree ⁪□^2^ I have no opinion ⁪□^3^ I disagree

37. What is your family’s feelings regarding your cigarette use? (Select ONE)

⁪□^1^ Friendly/Accepting

⁪□^2^ Normal/No specific reaction

⁪□^3^ Not Friendly/Non-accepting

□^4^ I don’t know

38. Which statement do you agree with the most?

⁪□^1^ Waterpipes are more addictive than cigarettes.

⁪□^2^ Cigarettes are more addictive than waterpipes.

⁪□^3^ Waterpipes and cigarettes are approximately the same in addiction.

39. Which statement do you agree with the most?

⁪□^1^ Waterpipes are more harmful than cigarettes.

⁪□^2^ Cigarettes are more harmful than waterpipes.

⁪□^3^ Waterpipes and cigarettes are approximately the same in harm.
