# Supplementary material for: Waterpipe smoking among health sciences university students in Iran: perceptions, practices and patterns of use
Source: BMC Res Notes. 2011 Nov 16;4:496. doi: 10.1186/1756-0500-4-496 (PMC3279519; doi:10.1186/1756-0500-4-496)
Supplement: Additional file 2 — Table 1 - Attitudes of waterpipe smoking between occasional and frequent waterpipe smokers. [file 1756-0500-4-496-S2.DOC]

**Tables**

## Table 1 - Attitudes of waterpipe smoking between occasional and frequent waterpipe smokers

| Attitudes of waterpipe smoking between occasional and frequent waterpipe smokers | | | | | |
| --- | --- | --- | --- | --- | --- |
|  | **n** | **Occasional** | **n** | **Frequent** | ***p*-value** |
| Healthiest way to use tobacco | 45 | 11.1% | 32 | 40.6% | 0.005* |
| Makes one look attractive | 45 | 4.4% | 34 | 8.8% | 0.65 |
| Dangerous to health | 45 | 73.3% | 34 | 67.7% | 0.62 |
| Relaxing | 42 | 26.2% | 32 | 62.5% | 0.002* |
| Gives me energy | 44 | 11.4% | 33 | 48.5% | 0.001* |
| Pleasant taste & smell | 45 | 51.1% | 34 | 73.5% | 0.06 |
| Part of my culture | 44 | 34.1% | 34 | 58.8% | 0.04* |
| Addictive | 45 | 22.2% | 34 | 20.6% | 1.00 |
| (*) denotes statistically significant result, (p < 0.05).  Results presented as percentage of question respondents who agreed with the statement. The total number of respondents (n) for each question varied. | | | | | |
